# Hospitals

**Published:** 1876-10

**Authors:** 


### Hospitals.

### SURGICAL DEPARTMENT, COUNTY HOSPITAL.

Service of Dr. Powell.

May 2, 1876. Dr. Powell presented and made remarks on the following cases:

1. *Scrofulous Abscess.*

A small spare man, middle aged. A lump the size of a child's head just below and external to the scapula; several weeks in coming. In the absence of all inflammatory symptoms there is good reason to conclude that it is simply a collection of matter there. Such abscesses are frequently called cold abscesses. Ill-clothed, and poorly fed people may have such collections. There is no need of a large opening. It is best simply to tap it and run a drainage tube through. It should occasionally be washed with some stimulating or disinfecting solution. Punctured, a stream of yellowish pus spouted forth like a fountain, half filling a quart basin.

2. *Encephaloid Cancer of the Left Thumb.*

An emaciated cachectic man, middle aged, a printer. His thumb got sore eighteen months ago. After trying everything, he had it cut off. It got well, but after six months the disease recurred. It is now as large as a man's fist—a pultaceous rosy mass. We should remember that if a tumor or any sort of sore recurs after having been removed, no matter how, we have something serious. Cancers recur. No non-malignant growth presents an appearance like that. The odor is quite characteristic; we may recognize it anywhere. The sooner that is removed the better; for cancer cells are being taken up; they will be carried on further and further until we shall have the entire system poisoned. The operation should go far away from the disease; then it will recur soon enough. Fortunately the glands in the axilla are not yet involved. The operation will be made just above the elbow. Esmarch's band must not be put on too low, lest we force up ill-conditioned pus.

1. *Fracture of Neck of Femur.*

May 5. An old man aged sixty-nine, just came in. He was injured yesterday by a fall from a sidewalk not more than three feet. He was picked up and carried home. Dr. P. proceeded to examine with reference to the nature of the injury, and first called attention to the expression of the limb. It was apparently a little bit shortened; there was a little fullness of the hip, and the foot was slightly everted. We are able to exclude all possible dislocations by just observing the limb. From the fact of the age, and his falling as he did, we are to suspect a fracture of the neck, for these fractures almost always occur in persons past sixty. Some surgeons say they can diagnosticate between fractures of the neck of the femur without and within the point of insertion of the capsular ligament. Nélaton used to say he would wait till the post-mortem. Sir Astley Cooper said he could not tell. We may exclude fracture of the shaft; beyond that it is, to say the least, a matter of extreme nicety. It will be well to speak with reserve as to the result in all these cases; for a fracture within the ligament is extremely inapt to unite. Those without, those partly without and partly within, and impacted fractures are disposed to unite. It is well always to say that probably it will not unite. It will be best to anæsthetize our patient. He will forever blame us for a bad result, and we shall forever have to face it. Dr. P. urged upon the class that they should know a good deal about fractures, saying that scarcely a month elapsed that some one did not come into the city with evidence of bad treatment or bad diagnosis. Measured from the ilium to the inner ankle, there was found to be half an inch of shortening. When the foot was taken in hand, there was pain produced in the hip and knee. The trochanter rotates. There was a slight grating sound detected. Dr. P. remarked that it was not necessary to go any further. The fracture was within the trochanter. The treatment in either case should be the same. But it should be remembered that any injury about the hip in an aged person, as a sprain, might result in interstitial absorption of the neck of the bone. The head would then seem to rest on the shaft. So that no matter how great the caution, the result of even slight injuries may simulate the result of a fracture. Simply apply extension. The ordinary adhesive plaster is not so good as that spread on Canton flannel. Afterward a webbing support will be put around the hip and a splint to roll the thigh inward. Twelve weeks will be required to remove these supports.

2. *Fracture of the Forearm, Middle Third, Caused by the Kick of a Horse.*

Laboring man, thirty. This fracture is as troublesome as any, except perhaps Colles' fracture, for there is so likely to result loss of pronation and supination. There is frequently thrown out a bony nodule between the bones. No surgeon can prevent that. In all such fractures of the forearm we simply want two splints, and we adapt them by making them a little wider than the arm. Set aside scientific or medical education and simply apply mechanical common sense. We want a splint that takes hold of the elbow and hand. The splints want to be exceedingly well padded with sheet batting, or what is better, folded strips from an old sheet, made to come well over all the corners. It is advisable to smear a little starch over it, if the patient lives at a distance, or is a little child. If there should be a tendency of either end of the bone to fall out of place, a little compress is required. The setting is simply to take hold and make it as near like the other limb as possible. Bandage lightly. At the end of two or three days look at it. Thenceforth if there is no deformity let it alone. Better leave the dressing on for six weeks.

1. *Fracture of Lower End of the Radius.*

May 9. An old lady fell on the stairs and struck the palm of her hand. The radial border of the forearm is seen to be shortened, drawing the hand to one side, and giving a prominence to the ulna. In an old person such a fall will generally produce a fracture of the radius. In a young person it will cause either a fracture or a dislocation of the elbow. We have a Colles' fracture. These fractures are exceedingly interesting from the difficulty of treatment. We are liable to have more or less deformity. Dress the hand just as the arm was dressed (May 5th). But there must be a large pad in front or there will be a projection forward. A little extension and moulding is all the setting it can receive. She will have to wear the splints five or six weeks, but she will come back a week from to-day. If any thing happens to prevent her return on that day, we shall have to send for her, for these cases must not be neglected, lest they fall into some unscrupulous hands and we be blamed for the deformity. There are more trials for malpractice with this and injuries of the elbow, than all other causes put together.

2. *Fracture of Forearm, dressed four days ago.*

Dr. P. remarked that he would remove the dressing to see if it was all right, although it could hardly be necessary yet. The position was as good as it could be; but a little compress might be needed on the outer surface of the arm, for there appeared to be a slight bowing upward. The compresses should be made of cloth, for that does not yield so much as batting, which may be used to line the splint.

3. *Injury of the Elbow.*

(April 25. Dr. Bogue.) Dressed at first with adhesive strips and pully, the weight of a brick being added. During the hurricane which unroofed the hospital the evening of May 6th, he betook himself to flight, carrying the brick with him. A good reason for not dressing these cases in the straight position is that the biceps is made taut. Only in fractures of the olecranon process is it admissible. Only fourteen days have elapsed since this injury, but it is fair to presume that the swelled muscles will act as splints. Hereafter all dressings will be left off. The arm will be bent and placed in a sling, and movement will be required each day to preserve as good a joint as possible.

4. *Paralysis of the Extensor Muscles of the Forearm and Hand following Convalescence from Fracture of the Humerus Middle Third.*

This case came in March 3d. It ordinarily takes longer for convalescence to become complete than for the bone to unite. The treatment here is passive motion effected by an india rubber cord attached at one end to the arm and having a loop at the other for the insertion of the fingers. He is instructed to resist the tension of the band. The paralyzed muscles are regaining strength by a kind of Swedish movement cure.

---

### NOTES OF CASES IN MERCY HOSPITAL.

Service of PROF. E. ANDREWS.

1. *Strangulated Hernia.*

Operation. The patient, a man aged thirty-five years, was admitted for mechanical obstruction of the bowels, which had existed fifty hours. A small inguinal tumor existed, but so insignificant that it was doubted whether it was the true cause of obstruction. Ether having been given, efforts were made to reduce it by taxis, but failed, as a similar attempt by Dr. W. Blanchard had done before admission. Prof. Andrews therefore proceeded to perform herniotomy, when a very small but tightly constricted portion of intestine was found, blacker in color than the average of strangulated intestines, but having no odor or feeling of grangrene, and apparently containing little or nothing which our aspirator could have withdrawn. The gut was probably forced through the ring in an empty state by some sudden and powerful action of the abdominal muscles. The stricture being cut, the intestine was returned, and the wound closed, and the patient made a rapid recovery. It is remarkable that this operation in Chicago has a much greater success than has been experienced elsewhere. Out of thirty-four cases only eight died, equal to twenty-four per cent., while in the Atlantic cities, and in Europe, the mortality is about fifty-four per cent. of all the cases. In the hospitals of Paris the mortality attains the frightful figure of sixty-eight per cent., which is probably due to the fact that septic diseases are less guarded against there, than in any other great hospitals in the world. In Chicago the patients rarely delay long in sending for help. With the usual wide-awake tendencies of a new city, they call a surgeon at once, and if an operation is needed it is done immediately at the house, so that these cases rarely appear in the hospitals. This promptness is one of the great causes of safety, as delay adds hourly to the danger.

2. *Colotomy.*

The patient, a woman, aged about thirty-six, was admitted for cancer high up in the rectum. The growth of the tumor had obstructed the gut so as to render defecation very difficult. Ether was given, and an attempt was made to rupture the cancerous stricture, but owing to its high location it could not be done with any force which it was safe to use. A week or two now elapsed, when it was found that the progress of the disease had rendered the obstruction nearly total. The abdomen was greatly distended, and the patient seemed rapidly failing from the effects of the obstruction. She was again anæsthetized, placed face downwards, with the abdomen across a large pillow. The colon was then exposed in the left lumbar region, behind the peritoneum by the usual incisions. The gut was emptied of its gas by a trocar, and then opened and stitched to the skin. No evil effect followed, and the relief from obstruction was complete. The cancer, of course, is not cured, but the patient is in comparative comfort, and does not experience any of the horrible inconveniences from the artificial anus which some authors describe. The orifice is prevented from leakage of gas and other contents by a water-proof pad. Gross discourages the operation, and thinks it better to let the patient die. Bryant, of England, on the other hand, says that the patients are surprisingly comfortable, and enjoy life well — a remarkable case of contradiction of opinion between two men eminent for sound judgment. The published statistics fail to show what is the real danger of the operation, because the compilers have included in one mass the patients who died of the operation itself and those who died afterwards of the cancers for which many of the operations were performed. This is a blunder which shows how infantile the important science of statistics still is. The operation is reckoned among the more difficult ones of surgery, but it is by no means overwhelmingly so, and when skillfully performed, is probably not very dangerous.

3. *Fracture of the Spine.*

Recovery. This patient received a fall, fracturing the dorsal region of the spine, and causing a visible projection at the point of injury. Fortunately the displacement was not sufficient to compress the spinal cord, and no paralysis occurred. He is recovering simply by being placed on his back in bed. Occasionally we see these cases of spinal fracture without injury to the cord. When they occur the patient usually recovers with little more trouble than if a limb were broken.

4. *Fracture of the Spine with Injury to the Cord. Death.*

This patient, aged about fifty, came in with fracture of the upper half of the dorsal region, total paralysis of the whole body below, and also fracture of the right thigh. Within a week he recovered partial sensation to the touch in the lower extremities, but not the motive power. Apparently the posterior columns of the cord were not entirely destroyed. He was placed on an air bed with extension on the broken thigh. Death occurred from general failure of health during the fourth week. There was no commencement of union in the fractured femur.

5. *Operation for Angular Deformity after Fracture.*

This patient came in with the upper third of the leg bent at an angle of twenty degrees in consequence of a former fracture. On being anæsthetized the union was too firm to yield to any reasonable force. A wedged-shaped piece was therefore sawn out of the tibia, and the fibula sawn across. The limb was then placed in a straight position and is now well advanced toward recovery.

6. *Abscess in the Medulla of the Femur; Consequent Caries of the Knee. Amputation of the Thigh.*

This patient entered with an inflamed knee, and a fistula discharging matter just above it. No dead bone could be reached by the probe. The knee rapidly became worse, and on being laid open was found carious. Being etherized, amputation was performed above the knee. On sawing across the shaft of the femur, the medullary cavity was found filled with pus, which extended five inches above the knee, where a growth of solid bone plugged the cavity and prevented effusion upward. Another section was taken from the femur in order to remove the whole of the disease. The outlet of the cavity was in front of the femur opposite the upper edge of the patella, from which point the pus burrowed into the joint. The patient is making a good recovery.

7. *Compound Dislocation of the Ankle. Resection of the Joint.*

Accidental violence drove the lower end of the tibia through the skin on the inner side, and the attending physician found it impossible to maintain a reduction. After some weeks the patient entered hospital with the end of the tibia still protruding from the wound. Ether was given, and the end of the bone exsected, when it was easily returned and retained. After a rather prolonged stay he was discharged, well advanced towards recovery.

